# A New Wrinkle in Hypodermic Medication

**Published:** 1882-09

**Authors:** E. C. Huse

**Affiliations:** Rockford, Ill.


### Domestic Correspondence.

### ARTICLE X.

### A NEW WRINKLE IN HYPODERMIC MEDICATION.

ROCKFORD, ILL., AUG. 6, 1882.

MESSRS EDITORS:—A somewhat troublesome abscess occurring to a patient recently under my care, with cervico-brachial neuralgia, has suggested a simple measure whereby this common and pestiferous result may be obviated.

Of however trifling import these abscesses may seem, instances are not wanting, where, in dilapidated or cachectic subjects, erysipelas, pyæmia, gangrene, or extensive sloughing have been their outcome. No "trifle" should ever be disregarded, in aught pertaining to the welfare of a patient.

The cause of these abscesses is doubtless always due to the introduction of some foreign body, however minute, along with the injection. In a word, the cause (exciting) is purely mechanical. The remedy is necessarily the same, and consists of a device to secure complete filtration of the injecta, destroying also bacteria and germs.

Placing a very small pledget of carbolized absorbent cotton into the upper cap of the syringe, which is readily done by unscrewing it from the tube, the liquid is introduced, the cap screwed on, and the air expelled by pointing it upward and slowly pushing the piston forward till the last bubble escapes. The needle may now be attached, and the injection made. The minutest particle of foreign material is thus inextricably entangled in the meshes of this improvised sieve, which in no case will interfere with the thread of the screw, and cannot, from its textile structure, clog or plug the caliber of the needle.

As a crucial test, the microscope failed to discover any shreds, fibers, granules or what not, after trying parts of several drops thus subjected to the filter.

It has occurred to me, as a sort of postulate, that wads of compressed absorbent cotton will be found far better than the inconvenient tablets lately used for the hypodermic syringe. The cotton may be made to absorb the solution, dried, and then compressed and packed in tubes like the tablets. A few minims of water, without special care in selection, may be drawn into the syringe, the upper cap unscrewed as above, the antiseptic absorbent cotton medicated wad filter introduced, the cap replaced and the gun fired. These tablets harden and become almost insoluble, even in hot water; with the wads this would be obviated, of course.

Very respectfully,

E. C. HUSE, M.D.

